# Contrast Enhancement of the Right Ventricle during Coronary CT Angiography – Is It Necessary?

**DOI:** 10.1371/journal.pone.0128625

**Published:** 2015-06-01

**Authors:** Madeleine Kok, Bas L. J. H. Kietselaer, Casper Mihl, Sibel Altintas, Estelle C. Nijssen, Joachim E. Wildberger, Marco Das

**Affiliations:** 1 Department of Radiology, Maastricht University Medical Center, Maastricht, The Netherlands; 2 CARIM Cardio Vascular Research Institute Maastricht, Maastricht University Medical Center, Maastricht, The Netherlands; 3 Department of Cardiology, Maastricht University Medical Center, Maastricht, the Netherlands; University of Bologna, ITALY

## Abstract

**Purpose:**

It is unclear if prolonged contrast media injection, to improve right ventricular visualization during coronary CT angiography, leads to increased detection of right ventricle pathology. The purpose of this study was to evaluate right ventricle enhancement and subsequent detection of right ventricle disease during coronary CT angiography.

**Materials and Methods:**

472 consecutive patients referred for screening coronary CT angiography were retrospectively evaluated. Every patient underwent multidetector-row CT of the coronary arteries: 128x 0.6mm coll., 100-120kV, rot. time 0.28s, ref. mAs 350 and received an individualized (P3T) contrast bolus injection of iodinated contrast medium (300 mgI/ml). Patient data were analyzed to assess right ventricle enhancement (HU) and right ventricle pathology. Image quality was defined good when right ventricle enhancement >200HU, moderate when 140-200HU and poor when <140HU.

**Results:**

Good image quality was found in 372 patients, moderate in 80 patients and poor in 20 patients. Mean enhancement of the right ventricle cavity was 268HU±102. Patients received an average bolus of 108±24 ml at an average peak flow rate of 6.1±2.2 ml/s. In only three out of 472 patients (0.63%) pathology of the right ventricle was found (dilatation) No other right ventricle pathology was detected.

**Conclusion:**

Right ventricle pathology was detected in three out of 472 patients; the dilatation observed in these three cases may have been picked up even without dedicated enhancement of the right ventricle. Based on our findings, right ventricle enhancement can be omitted during screening coronary CT angiography.

## Introduction

Previous studies have shown that multidetector-row computed tomography (MDCT) produces very high diagnostic image quality for a wide range of heart rates [1, 2]. Due to its high temporal resolution, not only evaluation of the coronary arteries, but also functional evaluation of the heart has become possible [1]. Takx et al. [3] reported that right ventricle (RV) morphology and function can be assessed during contrast enhanced CT of the heart. Quiroz et al. [4] found that RV enlargement deduced from CT images correlates with RV dysfunction seen on echocardiography.

Studies on RV function have been overshadowed by studies on left ventricular (LV) function [5], since the RV is generally considered to be affected by pathological processes affecting the cardiovascular system, and not a causal factor [6]. However, the RV is affected by, and contributes to, a number of disease processes, including not only acute pulmonary embolism (PE) and pulmonary artery hypertension (PAH), but also cardiomyopathy (CMP), RV ischemia or infarction, and tumour formation [6, 7].

Although RV assessment was shown to be feasible during CT angiography (CTA) of the heart, contrast timing has not yet been optimized for RV enhancement. This is due to the fact that patients are referred for CTA of the heart for suspected coronary artery disease (CAD) [8], and in this screening population RV pathology is not expected.

The purpose of this study was to evaluate if contrast media (CM) injection protocols, individually tailored for optimal RV enhancement during CTA of the heart, improve visualization and evaluation of the RV. If this does not prove to be the case, contrast bolus injection protocols can in future be tailored exclusively to coronary artery enhancement.

## Materials and Methods

### Ethics statement

Ethics approval and informed consent for the use of the images for this study was waived according to Dutch law. In the Netherlands, research covered by the Medical Research Involving Human Subjects Act must be submitted to an accredited medical ethics committee for approval. However, the Act does not include retrospective research using data from patient’s medical record or patient images. Therefore, our medical ethics committee (MEC) concluded that the research proposal of the current study does not, under Dutch law, require medical ethics approval, since there is no extra burden placed on research subjects (decision number: MEC 14-4-048). This study complies with the ethical principles of the Helsinki Declaration of 1964, revised by the World Medical Organization in Fortaleza, Brazil in October 2013.

All data regarding imaging and clinical data are digitally recorded for purposes of patient care in PACS systems and electronic patient records. Data for this retrospective analysis were anonymized beforehand; the key to this coding was determined by the coordinating radiologist (MD).

### Study population

509 consecutive patients referred for screening CTA of the coronary arteries between June 2012 and December 2012 were retrospectively evaluated. Patients were referred for coronary CTA (CCTA) from the cardiology outpatient department because of cardiac symptoms and suspected CAD [8] and no RV pathology was known beforehand. General exclusion criteria for contrast enhanced CCTA were: pregnancy, renal insufficiency (GFR <60), history of iodine contrast media reaction, inability to hold the breath during CTA, clinically unstable condition, unstable angina, and calcium scores >1000 found during scans without contrast.

37 of the 509 patients were excluded from CCTA: 32 due to a calcium score >1000; two due to known iodine allergy; two due to failure to obtain venous access; and one due to an inability to hold their breath during the scan. Thus, a total of 472 patients were included for retrospective image analysis.

### Scan and injection protocol

Every patient underwent MDCT of the coronary arteries using a 2^nd^ generation DSCT scanner (Somatom Definition Flash, Siemens Medical Solutions, Forchheim, Germany) with a 128x0.6 mm slice collimation; gantry rotation time of 280 ms; tube voltage of 100–120 kV depending on the individual patient size; tube current varied between 320–370 mAs_eff_. Different CT scan protocols were used. In patients with a stable heart rate of <60 beats per minute (bpm), a prospectively ECG-triggered “high pitch” spiral protocol was used (“Flash”-technique). In patients with a stable heart rate between 60-90bpm, a prospectively triggered “adaptive sequence” protocol was used (prospective sequential data acquisition). In patients with an irregular heart rate or a stable heart rate of >90bpm, a retrospectively gated “helical” protocol was used. A non-enhanced scan was performed to determine the coronary calcium score using the Agatston method [9].

Patients received 50 mg metoprolol tartrate orally (Selokeen, AstraZeneca, Zoetermeer, The Netherlands), two hours before CCTA. When indicated, an additional dose of 5–20 mg metoprolol tartrate was administered intravenously to lower the heart rate to <60 bpm. A maximum dose of 0.8 mg nitroglycerine (Isordil, Pohl-Boskamp, Hohenlockstedt, Germany) was given sublingually just prior to CCTA. Heart rate and ECG were monitored during CCTA.

Patients received an individualized (P3T Cardiac, and Stellant D, MEDRAD, Indianola, PA, USA) split-bolus of iodinated CM (iopromide 300 mgI/ml; Bayer Healthcare, Berlin, Germany), which allows for the adaption of injection rate, injection duration, and contrast volume for each individual patient. The software individually adapts both the volume (ml) of the main bolus and the mixed bolus (20% CM—80% Saline) as well as the flow rate (ml/s) based upon a nonlinear relationship between patient weight and duration of the CT data acquisition [10]. The individually tailored contrast split-bolus injection was followed by an intravenous saline flush of 40 ml. All injection related parameters were monitored using the Certegra Informatics Platform (MEDRAD, Indianola, PA, USA).

Image reconstruction was performed at 0.75 mm slice thickness with an increment of 0.5 mm using dedicated cardiac reconstruction kernel (Siemens B26f).

### Image analysis

**Fig 1:**
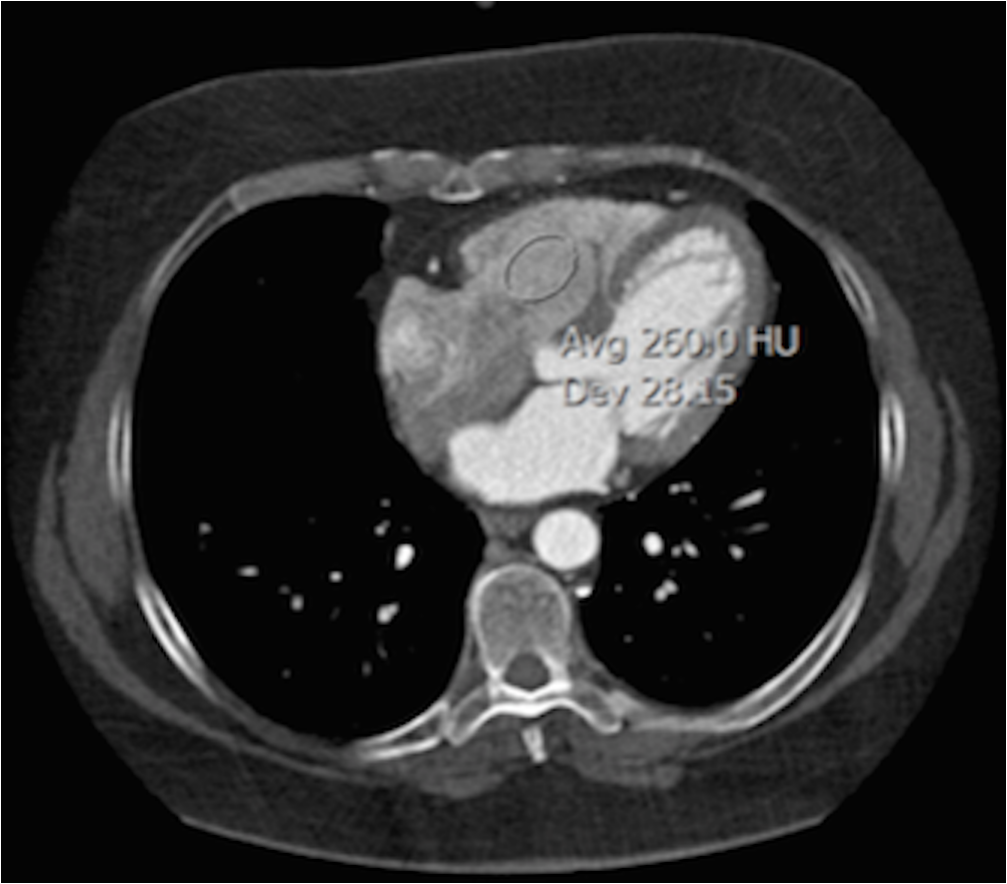
Contrast enhancement of the RV (260 HU) in CCTA.

Two experienced observers (a radiologist and a cardiologist), blinded to the injection technique, performed attenuation measurements in the RV using transverse sections on a medical workstation (IMPAX 6.5.2, Agfa Healthcare, Mortsel, Belgium). For the right chambers, measurements were performed in the centre of the RV. Regions of interest (ROI) were made as large as possible and placed centrally, in order to avoid the myocardium, valvular structures and papillary muscles **(Fig 1)**. Mean attenuation (in Hounsfield Units, HU) with standard deviation (SD) was measured in all 472 patients. Image quality was defined as the enhancement level within the RV cavity and this was subjectively based on how well the RV wall could be distinguished from the RV lumen: levels >200 HU were considered to be good image quality; levels between 140–200 HU were considered to be moderate image quality and levels <140 HU were considered to be poor image quality, because of the impossibility to distinguish the RV wall from its lumen and only general dilation might be detected. The total amount of CM used and peak flow rate were read from the data acquisition program Certegra Informatics Platform (MEDRAD). Finally, all 472 scan reports were analysed by the two observers for any pathology of the RV. In case of disagreement, consensus was reached by jointly reviewing findings.

### Statistical analysis

Baseline characteristics are expressed as medians (interquartile range) or percentiles. All other values are expressed as mean ± standard deviation (SD). Mean attenuation values, CM used and peak flow rates were measured and compared for statistical significance using the Independent-Samples Mann-Whitney U test, Tests of Normality being negative. To assess possible relationships between several covariates (e.g. CM bolus, flow rate, BMI and heart rate) and attenuation values, multivariable regression analysis was performed with RV enhancement (HU) as dependent variable.

Data analysis was conducted with SPSS version 20.0 (SPSS Inc, Chicago, IL, USA). All *p*-values are 2-sided, and *p-*values below 0.05 are considered statistically significant.

## Results

### Baseline Characteristics

Mean age was 58 (49–65); 246 (52%) of the included patients were men. Age, body mass index (BMI), and cardiac risk factors such as hypertension and high heart frequency, were recorded and are listed in **Table 1**.

**Table 1:** Baseline characteristics.

Baseline Characteristics	All patients (n = 472)
Age (years)	58 (49–65)
Gender (male)	246 (52%)
BMI* (kg/m^2^)	26 (24–30)
Hypertension	176 (37%)
Heart rate	62 (57–70)
Scan protocol	Flash 224 (47%) Adaptive 203 (43%) Helix 45 (10%)

*BMI = Body mass index

### CM bolus characteristics and RV analysis

From the 472 analysed patients, good image quality (>200 HU) was found in 372 patients and moderate (140–200 HU) and poor (<140 HU) image quality were found in 80 and 20 patients, respectively. Patients received an average CM bolus (mean±SD) of 108±24 ml and an average peak flow rate (mean±SD) of 6.1±2.2 ml/s. Mean enhancement of the RV of all patients was 268±102 (HU±SD). Pathology of the RV was found in only three of the 472 patients (0.63%); all three were diagnosed with RV dilatation. Mean enhancement of the RV was 282±79 HU is these three patients and this was not statistically different from the overall mean enhancement (*p* = 0.71). In addition, no significant differences were found in mean bolus nor mean peak flow between the three patients with RV dilatation as compared to other patients **(Table 2)**.

**Table 2:** Outcome.

	Flash Protocol (N)	Ad.Seq. Protocol (N)	Helix Protocol (N)	Mean bolus (ml±SD)	Mean peak flow (ml/s±SD)	Mean RV enhancement (HU±SD)
**Diagnostic images *(N = 472)***	224	203	45	108±24	6.1±2.2	268±102
**Dilatation *(N = 3 = 0*.*63%)***	2	1	0	109±22	6.4±1.2	282±79
*P*-value*				0.572	0.920	0.711

*p-value <0.05 was considered statistically significant

The results from multivariable regression analysis, incorporating CM bolus, flow rate, BMI and heart rate with RV enhancement as dependent variable, are stated in **Table 3**. The parameters CM bolus, BMI and heart rate were found to be negatively associated with RV enhancement and this association was only significant for the parameter ‘heart rate’ (p<0.02). The parameter ‘flow rate’ showed—as only one—a positive association with RV enhancement, however, this was not significant (p>0.33).

**Table 3:** Multivariable regression.

	RV enhancement	*P*
***Constant***	*298*.*89*	*0*.*001*
***Variables***		
CM* bolus	-0.46	0.372
** Flow rate**	11.20	0.339
** BMI**	-1.50	0.258
** Heart rate**	-0.07	0.018

Multivariable regression analysis with RV enhancement (HU) as dependent variable. The regression constant for RV enhancement and the regression coefficients for the covariates are given.

*CM: contrast media.

## Discussion

CCTA is currently the golden standard for evaluation of the coronary arteries. Yet it is unclear whether RV pathology will be picked up during CCTA in a screening population.

With respect to the prevalence in the general population, hypertrophic cardiomyopathy (HCM) occurs in approximately 1:5000 [11, 12] and the estimated prevalence of arrhythmogenic right ventricular cardiomyopathy (ARVC) is 1:5000 as well [13]. The reported prevalence of primary cardiac tumors is only 0.002–0.03% [14–16]. This implies that RV pathology will hardly be detected, especially using CCTA in a screening population. This was also confirmed by the current study, as only three of the investigated patients with symptoms of CAD were diagnosed with RV pathology.

Assessment of ventricular size, mass, and function (e.g. ejection fraction and wall motion) are important factors in diagnostic imaging with respect to the detection of RV pathology. For evaluation of RV and LV volume and function, cardiac magnetic resonance (CMR) is currently considered to be the reference standard [17–19]. However, Takx et al. [3] evaluated the accuracy of LV and RV function and myocardial mass based on a dual-step, low radiation dose prospectively ECG-triggered protocol (adaptive sequence), using CMR as a reference standard. They concluded that CT measurements correlated well with those obtained using CMR.

In the current study, helical protocols as well as adaptive sequence protocols might have been useful to assess global RV function. For example, functional evaluation of the heart during the entire cardiac cycle (at each 10% of 0–100%) is feasible for helical protocols. However, for adaptive sequence there are only two reconstructed phases of the cardiac cycle (best systolic and best diastolic) available to evaluate ventricular function. As evaluation of the cardiac function is no part of the standard evaluation of a CCTA and this was a retrospective study, these parameters were not assessed.

Besides the choice of an optimal scan protocol for evaluation of the RV, an optimized injection protocol is of importance as well. Optimization of injection parameters is necessary during CTA, in particular for cardiac applications in order to ensure high, homogeneous and consistent vascular attenuation and minimisation of artefacts [20–23]. Several different injection protocols with special emphasis on high and homogeneous attenuation of coronary arteries and the right side of the heart have been evaluated and compared [10, 24, 25]. However, due to modern power injector systems with minimized CM volumes and improved scan timing techniques, the saline chaser method has become so effective that there is hardly any CM left in the RV at the time of scan acquisition in many patients [24]. As a consequence, right heart diseases cannot be detected, right heart structures cannot be assessed, and pathological thickening of the RV myocardium and intraventricular septum cannot be appropriately evaluated. Thus, sufficient attenuation of the right heart is needed in order to derive parameters of right ventricular function from CT scans [4, 26].

Nance et al. [25] evaluated four different CM injection protocols and concluded that CM delivery protocols with high iodine concentration and high iodine delivery rate (IDR) provide the best image quality of the lung, through high attenuation in the target tract (RV and pulmonary arteries). Kerl et al. [24] retrospectively compared a split-bolus CM injection protocol to a biphasic and a monophasic protocol in terms of visualization of right and left heart. They concluded that a split-bolus injection protocol was the first choice for visualization of the RV. Seifarth et al. [10] compared individually tailored split-bolus protocols to conventional injection protocols using fixed injection parameters and found best quality images were obtained when using an individual tailored CM bolus. However, they focused mainly on the coronary arteries, and not the RV.

And although RV visualisation could be improved by using (individualized) split-bolus contrast injection protocols [24], there is still no evidence that this has any clinically relevant consequences as RV pathology was uncommon in our study population and is uncommon as well according the general prevalence. On the other hand, if the scan protocol is focussed on the evaluation of the right heart—in patients who are suspected for RV pathology—the use of individualized contrast injection protocols could be beneficial.

**Fig 2:**
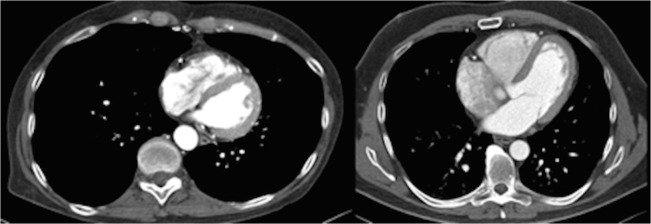
Contrast enhancement patterns of the enlarged right ventricle with optimal enhancement of the left ventricle in both cases.

In this study, the only pathology found was RV dilatation, the diagnosis of which could have been made without optimal enhancement of the RV: RV enlargement will normally stand out when there is optimal LV enhancement **(Fig 2)**.

During the individualized split-bolus contrast injections of this study a large amount of CM (108±24ml) was used. This is disadvantageous for patients with a history of iodine intolerance, and might increase the risk of contrast induced nephropathy (CIN) [27]. Using our standard injection protocol with a fixed volume of 75ml, the volume could be reduced by 30% compared to the individualized injection protocol, but at the expense of optimal RV enhancement. The difference in RV enhancement between individualized and standard injection protocols could be contributed more likely to the fact that the standard protocol consists of a single bolus instead of split-bolus rather than differences in CM volume, as no correlation was found between CM volume and increased RV enhancement in this study. Therefore, the use of minimal iodine should be considered during routine clinical scanning.

There are limitations to this study, and one of them concerns the study population. Every patient who received a screening CTA of the coronary arteries was evaluated, but no background information pertaining to the presence of thoracic disease, with potential impact on the RV, was available. Additionally, based on the prevalence of RV pathology, a larger group of patients (n = 5000) should be investigated to detect any pathology.

## Conclusion

We were not able to provide evidence that enhancement of the RV during screening CCTA is beneficial, as we hardly detected any RV pathology. Potentially, therefore, contrast bolus could be reduced and exclusively tailored to enhancement of the coronary arteries.
